# Spontaneous deep vein thrombosis of the upper arm due to an intravascular myopericytoma: A case report and literature review

**DOI:** 10.1097/MD.0000000000036566

**Published:** 2023-12-08

**Authors:** Lee Chan Jang, Kwon Cheol Yoo

**Affiliations:** a Department of Surgery, Chungbuk National University Hospital, Chungbuk National University, Cheongju, South Korea.

**Keywords:** case report, intravascular myopericytoma, spontaneous deep vein thrombosis, upper arm

## Abstract

**Introduction:**

Intravascular myopericytomas are a rare type of myopericytomas. In most previously reported cases, these were benign, occurred on the legs or neck, and had low recurrence rates. We have described a unique case of an intravascular myopericytoma that caused spontaneous deep vein thrombosis.

**Main symptoms, important clinical findings, and main diagnoses:**

A 37-year-old man presented with sudden-onset pain and swelling in the upper arm; physical examination revealed a 10 cm, palpable, firm, and mobile lesion in the upper arm. A biopsy revealed intravascular myopericytoma; immunohistological examination revealed a lesion in the lumen of the basilic vein. The tumor comprised abundant myxoid stroma with spindle cells proliferating in a concentric perivascular manner around the blood vessel. The tumor cells stained positive for CD34 and smooth muscle actin.

**Therapeutic interventions and outcomes:**

The patient underwent total excision of the mass under local anesthesia; no recurrence was observed thereafter. A literature review was performed using PubMed and Google Scholar; the key terms were “intravascular myopericytoma” and “IVMP.” Nineteen cases of intravascular myopericytomas across 14 articles published between January 2002 and January 2022 were identified. These involved 11 men and 7 women (sex was unknown in 1 case); the ages were 22 to 80 years (mean: 59.8 ± 14 years). In most cases, the tumor was slow-growing, and the etiology was previous surgical history or trauma. No pain was reported by patients with tumors on the face or feet, and no recurrence was observed after surgery in any of the reported cases. Immunohistochemical staining for smooth muscle actin, h-caldesmon, calponin, and CD34 was performed for differential diagnosis. Contrary to the slow-growing nature reported in the literature, the nature related to growing in the present case was unclear that lesion was discovered because of sudden pain caused by thrombosis. However, the diagnostic method and recurrence rate in our case were similar to those in the previously reported cases.

**Conclusion:**

Our case shows that although intravascular myopericytomas are rare, they can cause spontaneous thrombosis. They have low recurrence rates after complete resection. Spontaneous deep vein thrombosis that occurs in rare locations must be treated after determining the causes.

## 1. Introduction

Myopericytomas, first described by Granter et al^[1]^ in 1998, are rare tumors. In 1992, Dictor et al^[2]^ defined myopericytes as transitional cells between smooth muscle cells and pericytes in the blood vessels. Myopericytoma was officially classified as a subtype of perivascular tumors by the World Health Organization in 2002.^[3]^ Although several reports and literature reviews on myopericytomas have been published, <20 cases of intravascular myopericytomas have been described since the first report by McMenamin and Calonje in 2002.^[4]^ In this report, we have presented a case of an intravascular myopericytoma in the upper arm that was discovered because of the occurrence of spontaneous thrombosis due to luminal narrowing caused by intravascular myopericytoma. We have also presented our findings from a literature review of previously reported cases.

## 2. Patient information

A 37-year-old man visited our outpatient clinic with complaints of pain and swelling in the upper arm, which developed suddenly 1 day before presentation.

The patient used to take antithrombotic drugs for excessive obesity and cardiac hypertrophy in the past but was not taking them currently. To lose weight, he had undergone a gastric sleeve resection 4 months prior to presentation, which resulted in a weight loss of approximately 30 kg; he had maintained this. The patient denied any history of trauma.

## 3. Clinical findings

**Figure 1.**
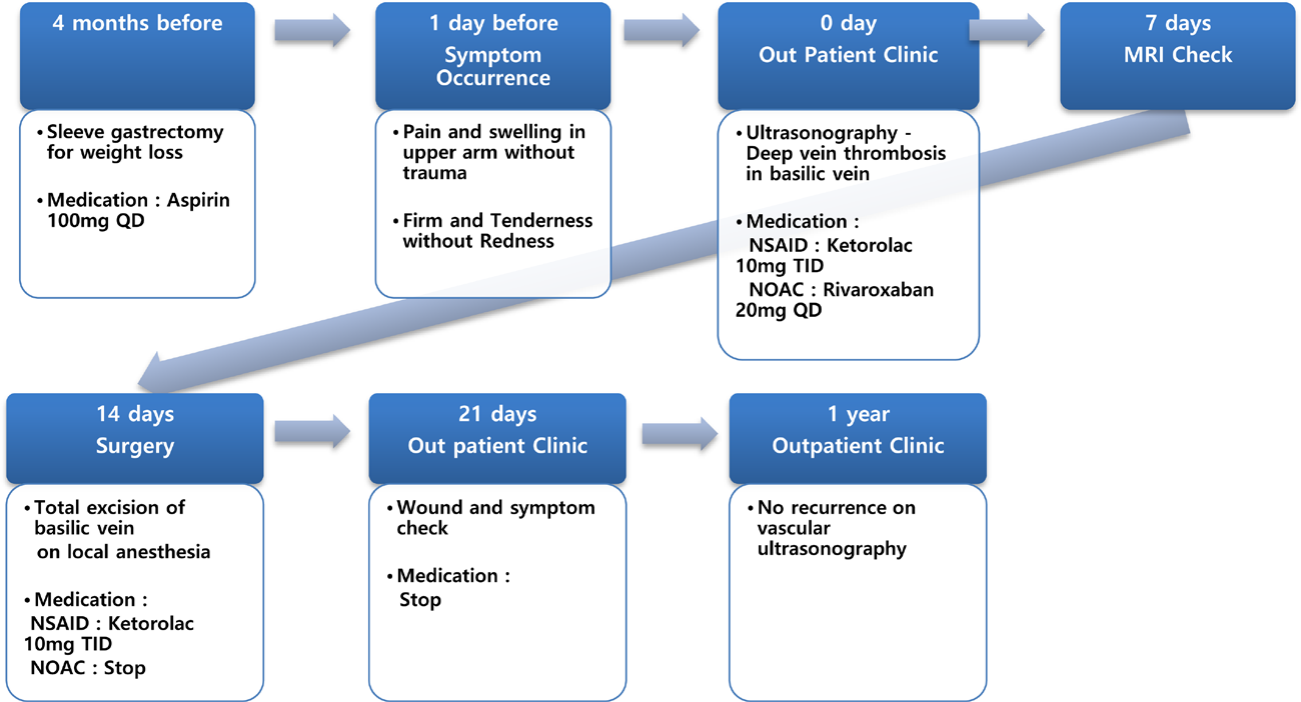
Timeline of the case.

Grossly, a non-erythematous lump was observed on the inner side of the upper arm. It was tender upon palpation during physical examination and was found to be a firm but slightly movable mass-like lesion (approximately 10 cm in size). These observations suggested an intravenous thrombus rather than a lipoma or cyst. The timeline of the case is shown in Figure 1.

## 4. Diagnostic assessment and therapeutic intervention

**Figure 2.**
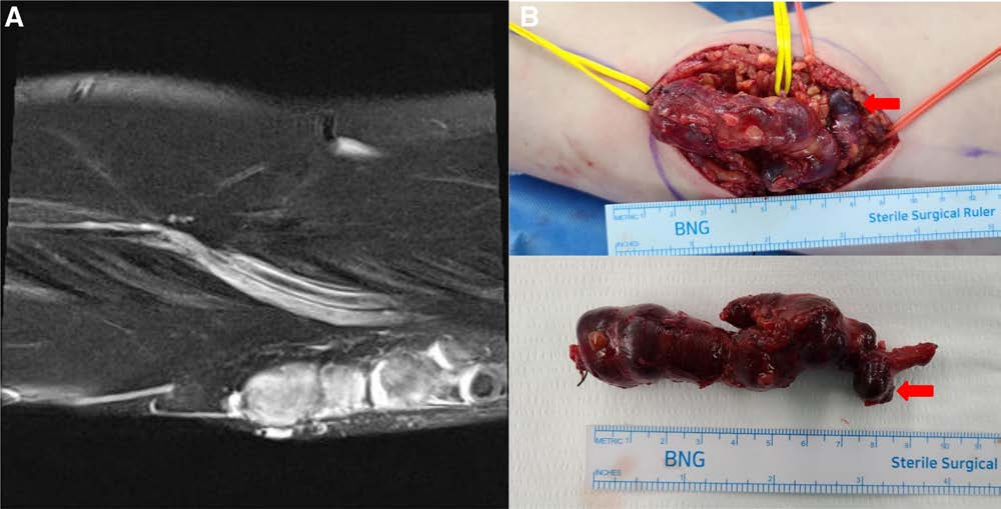
Preoperative radiograph (A) and intraoperative photographs (B) of right upper arm. (A) Magnetic resonance image of the upper arm shows venous enlargement with a thrombus in the basilic vein. (B) Operative findings that intravascular myopericytoma caused occlusion and venous enlargement by narrowing the proximal basilica vein (red arrow).

**Figure 3.**
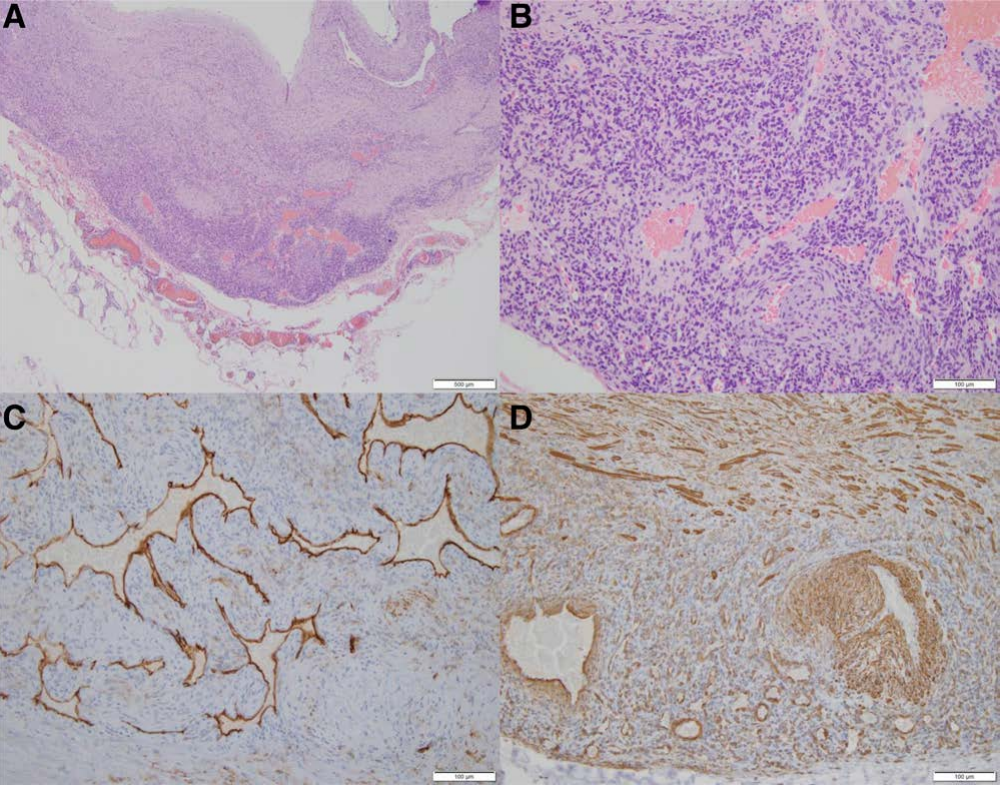
Histopathologic findings of intravascular myopericytoma (A–D). (A) The tumor is composed of abundant myxoid stroma and blood vessels (hematoxylin and eosin [H&E], ×40). (B) The concentric perivascular proliferation of bland, oval-to-spindle cells shows multi-layered enveloping of the blood vessels (H&E, ×100). (C) Focal positive immunostaining for CD34 (CD34, ×200). (D) Diffusely positive immunostaining for smooth muscle actin (SMA, ×200).

Ultrasonography was performed to differentiate between potential causes of the lump, such as lipomas and cysts. Magnetic resonance imaging, performed for a more accurate diagnosis, revealed that the basilic vein was enlarged and filled with a thrombus (Fig. 2). Non-vitamin K antagonist oral anticoagulants were administered preoperatively to prevent aggravation of deep vein thrombosis. Total excision of the vessel was performed under local anesthesia. Intraoperatively, the basilic vein was noted to be filled with a thrombus measuring 10 × 4 cm, with abnormal thickening of the vessel wall. After excision, no remnant thrombus was seen in the surgical field; the non-vitamin K antagonist oral anticoagulants were discontinued, and a non-steroidal anti-inflammatory drug was prescribed for pain control for approximately 7 days. The excised tissue was fixed using 10% buffered formalin for paraffin embedding. Paraffin sections of the specimen were stained with hematoxylin and eosin. The antibodies used for immunohistochemical staining were S-100 protein, anti-smooth muscle actin, CD34, and anti-desmin antibodies (Fig. 3).

The tumor (constituting the entire vessel wall) was visible on hematoxylin–eosin-stained sections (×400) during a histopathological examination. Concentric perivascular proliferation of bland, oval-to-spindle cells was observed along with multi-layered enveloping of the blood vessel (hematoxylin–eosin stain, ×200). Immunohistochemical staining was negative for S-100 (positive in neurogenic tumors), desmin (positive in myogenic tumors), and smooth muscle actin and CD34 (diffusely and focally positive in myeloid cell-derived tumors, respectively).Thus, the patient was diagnosed with an intravascular myopericytoma based on the histological examination results.

## 5. Follow-up and outcomes

The patient recovered well and experienced no complications, such as pain, in the first 7 post-operative days. Subsequently, all medications administered during treatment were discontinued. No recurrence was observed on physical examination or ultrasonography at 1 year after the surgery.

## 6. Literature review

We conducted a literature search in PubMed and Google Scholar using the key terms “intravascular myopericytoma” and “IVMP.” This yielded 14 case reports on intravascular myopericytomas published between January 2002 and January 2022; 1 of these was also mentioned in a literature review on myopericytomas. Although histopathological, etiological, and recurrence data were not available in the literature, we were able to collect information on patient sex, age, and location.

## 7. Discussion

In 1942, Stout and Murray^[5]^ proposed that pericytes (perivascular modified smooth muscle cells) are the origin of hemangiopericytomas. Their theory was verified by studies analyzing the structural patterns of these tumors,^[6–8]^ but not fully supported by immunohistochemical findings. Staining has been performed nonspecifically for vimentin and CD34, but not for actin or other myoid markers.^[8–10]^ On the basis of their clinical features, hemangiopericytomas are classified as adult hemangiopericytomas, sinonasal hemangiopericytomas, infantile hemangiopericytomas, and myopericytomas.^[11]^ Myopericytomas are closely related to myofibromas, glomus tumors, and angioleiomyomas; however, their distinctive feature is the presence of perivascular, concentric, multi-layered, oval-to-spindle cells.^[1]^ Most of these tumors are benign^[12,13]^; however, multiple malignant tumors may develop and have been found to be related to the acquired immunodeficiency syndrome.^[14,15]^

The main limitation of the present case report is that it does not present any unique laboratory values; however, it has described a case involving spontaneous deep vein thrombosis, a previously unreported clinical feature of intravascular myopericytomas. In this report, the post-surgery follow-up period was approximately 1 year. Therefore, recurrence could not be confirmed through an extended follow-up period. The misdiagnosis and treatment of this condition as simple deep vein thrombosis rather than deep vein thrombosis caused by IVMP (in cases of incomplete surgical resection or receiving only antithrombotic treatment), might lead to potential recurrence. Therefore, this case emphasizes the necessity of confirming the diagnosis through complete surgical resection and a pathological biopsy.

Based on our literature review, this is the first case of a myopericytoma presenting with thrombosis, which was discovered incidentally and without any history of trauma or surgery. In the basilic vein where the intravascular myopericytoma occurred, a venous aneurysm was formed due to the obstructed blood flow; this likely induced the thrombosis.

As shown in Table 1, 19 cases of myopericytomas have been reported previously. Mentzel et al^[15]^ conducted a histopathological retrospective review of myopericytomas and found that the mean age of the patients was 59.2 ± 14 years, with slightly more cases reported in men than in women (11 vs 6). Compared to myopericytomas, which occur at an average age of 52 years, intravascular myopericytomas appear to occur at an older age.^[15]^ However, intravascular myopericytomas most frequently occur in the extremities (especially lower extremities), followed by the neck and head; thus far, the present case is the only 1 involving the upper arm.

**Table 1:** Summary of previous reported intravascular myopericytoma.

	Authors/year	Age	Sex	Site	Complaints	Etiology	Onset	Recurrence	Stain positivity
1	McMenamin/2002	54	Male	Thigh	Slow growth with pain	Previous tumor excision	10 yr	None	SMA (Diffuse +)/CD34 (Focal +)
2	Mentzel et al/2006	71	Male	–	–	–	–	–	–
3		57	Male	Hand	–	–	–	–	–
4		80	Female	Trunk	–	–	–	–	–
5		22	Male	Thigh	–	–	–	–	–
6		62	Male	Foot	–	–	–	–	–
7	Ide et al/2007	46	Female	Oral mucosa	Slow growth without pain	Previous tumor excision	2 yr	None	SMA/h-caldesmon
8	Woollard et al/2007	63	Male	Palm	Swelling with pain	–	–	–	–
9	Laga et al/2008	64	Male	Nose/Forehead	Slow growth without pain	Pervious surgery	8 mo	None	SMA (Diffuse +)/Calponin (Focal +)
10		72	Female	Oral Gingiva	Slow growth without pain	Previous dental care	–	None	SMA/h-caldesmon
11	Park et al/2010	79	Female	Infraorbital	Slow growth without pain	Previous tumor excision	5 yr	-	SMA/Masson-trichrome
12	Rullier et al/2010	71	–	Palm	Swelling with pain	–	5 yr	None	SMA/h-caldesmon
13	Ko et al/2011	67	Male	Thigh	Slow growth with pain	–	15 yr	-	SMA (Diffuse +)/CD34 (Focal +)
14	Mahapatra et al/2014	59	Female	Finger	Slow growth with pain	–	2 yr	None	SMA/h-caldesmon
15	Xia et al/2015	50	Male	Shin bone	Pain	Trauma	5 yr	None	SMA/h-caldesmon
16	Valero et al/2015	48	Male	Heel	Slow growth without pain	–	15 mo	None	SMA/h-caldesmon
17	Agustí et al/2016	63	Female	Foot	Slow growth without pain	Previous tumor lesion	–	None	SMA/h-caldesmon
18	Calleros et al/2016	38	Female	Neck	Slow growth with pain	–	5 yr	None	Vimentin/Calponin/CD34
19	Kagoyama et al/2020	71	Male	Foot	Slow growth with pain	Repeated trauma	30 yr	None	SMA/h-caldesmon

SMA = smooth muscle actin.

Myopericytomas are not accompanied by pain; however, most intravascular myopericytomas are characterized by slow growth (8 months–30 years) and pain, which are reported in about half of the cases.^[4,16–22]^ Tumors that occur on the face and feet have rarely been reported to be painful.^[23–27]^ McMenamin and Calonje^[4]^ assumed that pain is caused by a tumor thrombus, as in our case. Furthermore, in all the previously reported cases, the etiology has been attributed to previous surgical manipulation or trauma.^[15,20,22,24,25,27]^

In most cases, immunohistochemical staining was performed for smooth muscle actin, followed by h-caldesmon. CD34, calponin, vimentin, and Masson trichrome staining were rarely performed. Because h-caldesmon stains smooth muscle cells and calponin stains actin filaments, they are used to identify tumors originating from smooth muscle cells.^[28]^ Vimentin is used to identify tumors of mesenchymal origin, but is not widely used due to its non-specificity. CD34 distinguishes hemangiopericytomas (positive staining) from endometrial stromal sarcomas (negative staining).^[29]^ In our case, staining was performed for smooth muscle actin and CD34 (common markers used for diagnosis); staining for desmin and S-100 was performed to rule out tumors of other origins.

In all cases of intravascular myopericytomas in the literature as well as in our case, the tumors were diagnosed as benign, and no recurrence occurred after surgical resection. These observations were contrary to those in previously reported cases of myopericytomas in general, wherein recurrence occurred in 5% to 10% of the cases.

Our report identifies spontaneous deep vein thrombosis as a rare complication of intravascular myopericytomas, which may help in their detection. These tumors have low recurrence rates, and complete resection is an effective treatment.

## 8. Patient perspective

The patient was satisfied that they had no recurrence and no discomfort, except for mild postoperative pain.

## Acknowledgment

We would like to thank Editage for English language editing.

## Author contributions

**Conceptualization:** Kwon Cheol Yoo.

**Data curation:** Kwon Cheol Yoo.

**Formal analysis:** Kwon Cheol Yoo.

**Investigation:** Lee Chan Jang.

**Methodology:** Lee Chan Jang, Kwon Cheol Yoo.

**Project administration:** Kwon Cheol Yoo.

**Supervision:** Lee Chan Jang.

**Writing – original draft:** Kwon Cheol Yoo.

**Writing – review & editing:** Kwon Cheol Yoo.
